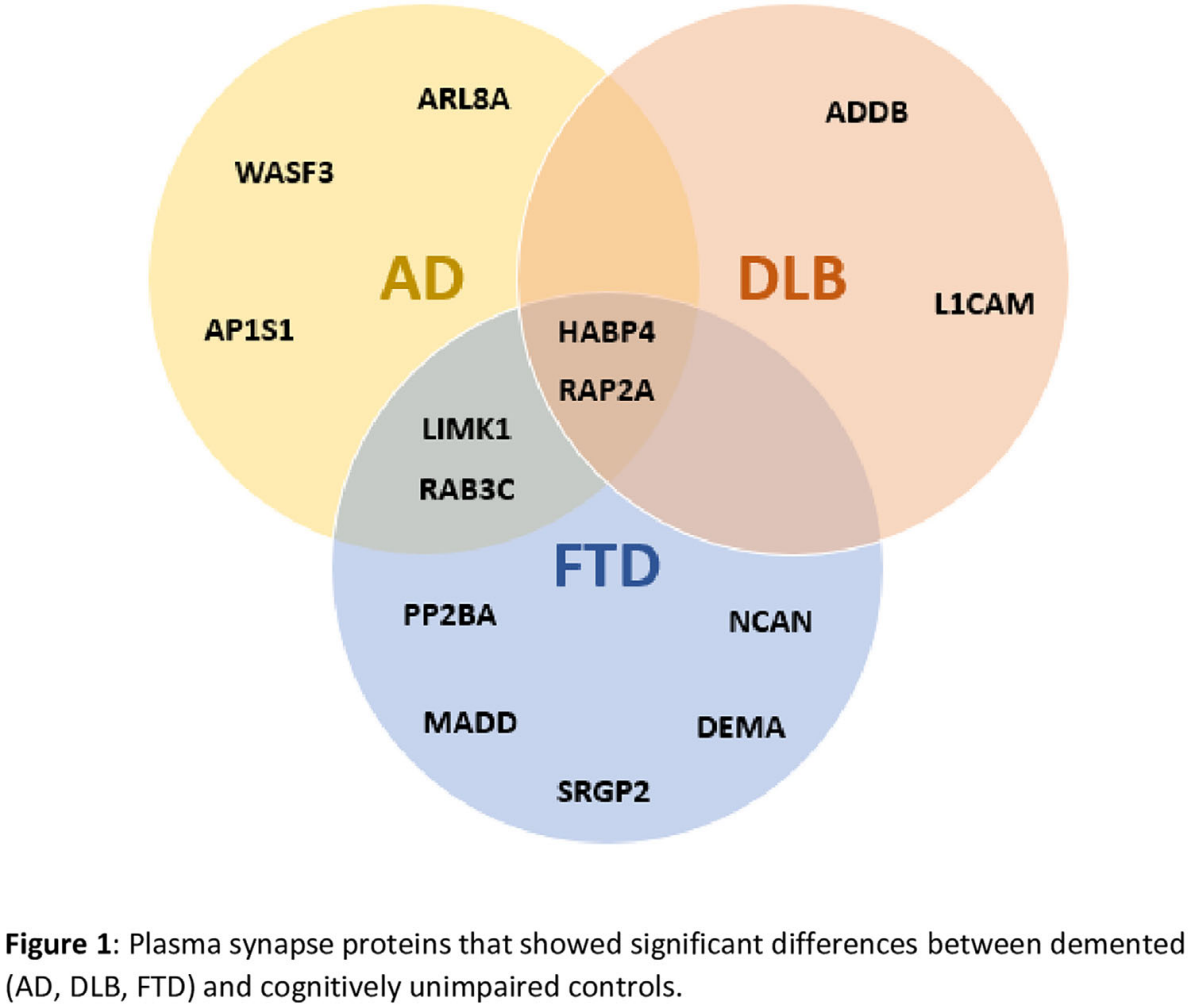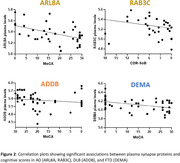# Plasma synapse proteins discriminate between AD, DLB, and FTD dementias and closely predict memory dysfunction

**DOI:** 10.1002/alz.094710

**Published:** 2025-01-09

**Authors:** Raquel N Taddei, Pia Kivisäkk, Matthijs B de Geus, Charles N Klein, Henrik Zetterberg, Teresa Gomez‐Isla, Steven E Arnold

**Affiliations:** ^1^ Massachusetts General Hospital, Harvard Medical School, Boston, MA USA; ^2^ University College London, Dementia Research Institute, London United Kingdom; ^3^ Clinical Neurochemistry Laboratory, Sahlgrenska University Hospital, Gothenburg Sweden

## Abstract

**Background:**

Robust biomarkers are urgently needed to detect, diagnose, and predict memory decline in the three most common neurodegenerative dementia syndromes, Alzheimer disease (AD), dementia with Lewy bodies (DLB), and frontotemporal dementia (FTD). The currently used diagnostic and therapeutic compounds target the disease‐defining neuropathologic signatures (amyloid, tau, alpha‐synuclein, TDP43) but show limited efficacy. Growing evidence suggests that synapses form the anatomical basis of cognition and that synapse dysfunction closely predicts dementia onset and progression in these dementing disorders. Yet, the plasma synapse signatures characterizing the three most common neurodegenerative dementia syndromes remain unexplored.

**Method:**

We analyzed plasma samples from 100 individuals with autopsy‐confirmed neurodegenerative dementias (24 AD, 30 DLB, 30 FTD) and 16 controls without dementia or neuropathological abnormalities enrolled in the Massachusetts ADRC longitudinal cohort. Samples were obtained ≤3 years prior to death and analyzed using unbiased mass‐spectrometry (Proteograph^TM^ Seer Inc.). Plasma proteins were cross‐referenced with the SynGO database to select synaptic, and with the Human Protein Atlas to include brain‐expressed proteins. ANOVAs, Bonferroni corrections, and t‐tests were applied using R functions on log‐transformed data. Linear correlation analyses evaluated associations of synapse protein levels and cognitive scores (MoCA, CDR‐global, CDR‐SoB) in each dementia syndrome.

**Result:**

We detected >80 synaptic proteins in the plasma of the 100 cases. A subset of 14 plasma synapse proteins significantly differed between dementia cases and controls. Some synapse proteins were increased across dementia syndromes (HABP4, RAP2A), while subsets of them were selectively altered in only one specific neurodegenerative condition (ARL8A, WASF3, AP1S1 in AD; ADDB, L1CAM in DLB; SRGP2, PP2BA, MADD, DEMA, NCAN in FTD). Significant correlations with memory scores were found for some synapse proteins (ARL8A, RAB3C in AD, ADDB in DLB, and DEMA in FTD).

**Conclusion:**

Synapse‐associated proteins are altered in the plasma of AD, DLB, and FTD, compared with non‐demented control cases. Select synapse protein signatures may serve as biomarkers to detect global cognitive dysfunction in various dementing diseases, and to accurately discriminate between dementia syndromes. The future investigation and validation of plasma synaptic biomarkers may provide an unprecedented tool for the detection and differential diagnosis of neurodegenerative dementia syndromes in research and clinical settings.